# Supplementary material for: Thermoelectric signature of quantum critical phase in a doped spin-liquid candidate
Source: Nat Commun. 2023 Jun 21;14:3679. doi: 10.1038/s41467-023-39217-7 (PMC10284844; doi:10.1038/s41467-023-39217-7)
Supplement: Supplementary file 1 — Supplementary Information [file 41467_2023_39217_MOESM1_ESM.pdf]

Supplementary Information for

Thermoelectric signature of quantum critical phase in a doped spin liquid candidate

K. Wakamatsu[1], Y. Suzuki[1], T. Fujii[2], K. Miyagawa[1], H. Taniguchi[3] and K. Kanoda[1,4,5,6]*

[1]*Department of Applied Physics, University of Tokyo; Bunkyo-ku, Tokyo, 113-8656, Japan.*

[2]*Cryogenic Research Center, University of Tokyo; Bunkyo-ku, Tokyo, 113-0032, Japan.*

[3]*Graduate School of Science and Engineering, Saitama University; Saitama 338-8570, Japan.*

[4]*present address: Max Planck Institute for Solid State Research; Heisenbergstrasse 1, 70569 Stuttgart, Germany*

[5]*present address: Physics Institute, University of Stuttgart; Pfaffenwaldring 57, D-70569 Stuttgart, Germany*

[6]*present address: Department of Advanced Materials Science, University of Tokyo; Kashiwanoha 5-1-5, Kashiwa 277-8561 Chiba, Japan*

Supplementary Note 1. Crystal structure and band filling

Most κ-type BEDT-TTF compounds have the composition of κ-(BEDT-TTF)$_2X$ with anion, X. κ-(BEDT-TTF)$_2X$ has a quasi-two-dimensional structure with conducting BEDT-TTF layers and insulating X layers alternately stacked. In the conducting layer, BEDT-TTF dimers form an isosceles triangular lattice characterized by two different transfer integrals, t and t'. In the insulating layer, anion, X, electronically has a closed shell structure with a valence of -1. Therefore, the valence of BEDT-TTF is +0.5; namely, one hole resides per one dimer site so that the band is half-filled in most κ-(BEDT-TTF)$_2X$ systems.

κ-(BEDT-TTF)$_4$Hg$_{2.89}$Br$_8$ is exceptional in that the lattice periodicity of Hg ions in the insulating layer is incommensurate with that of BEDT-TTF molecules in conducting layers[1,2], resulting in a deviation from the half filling of the band[3]. The incommensurability, namely, the nonstoichiometry in κ-(BEDT-TTF)$_4$Hg$_{2.89}$Br$_8$ is not varied but fixed by chemistry to give 11% hole doping to half-filing. Note that mercury-included compounds often have incommensurate structures[4].

The ratio, t'/t, in κ-HgBr is 1.02 according to Ref.[5], where the t and t' values are evaluated by tight-biding approximations based on the extended Hückel calculations of molecular orbitals.

Supplementary Note 2. Temperature dependence and contour plot of -*S*/*T* of samples #1 and #2

We measured $-S/T$ for two separate samples #1 and #2. Supplementary Figures 1a and 1b show the $-S/T$ data for the two samples separately and Supplementary Figures 1c and 1d show their contour plots on the temperature-pressure plane. The results of the two samples coincide with each other.

Supplementary Note 3. Temperature dependences of the resistivity and -*S*/*T* at 0.4 and 1.5 GPa

Supplementary Figures 2a and 2b show the temperature dependences of $-S/T$ and the resistivity of sample #2. The resistivity is normalised to the value at 15 K. The Seebeck coefficient and resistivity were measured simultaneously at the same setup. At 1.5 GPa, the resistivity shows the squared temperature dependence and $-S/T$ is nearly constant at low temperatures, confirming the Fermi liquid behaviour. At 0.4 GPa, the resistivity shows linear-in-temperature dependence down to T_c and $-S/T$ exhibit the logarithmic divergence down to T_c.

Supplementary Note 4. Definition of T_c and T^*

Supplementary Figure 3 shows the temperature dependence of $-S/T$ at zero field under 0.3 GPa for sample #1. The uppermost and lowermost values and the midpoint defined in Fig. 3b correspond to the upper and lower bounds of the error bar. The onset temperature, T^*, is defined as the temperature at which $-S/T$ begins to deviate from the high-temperature normal-state behaviour.

Supplementary Note 5. Pressure dependence of |γ'| and T_c

Supplementary Figure 4 shows the pressure dependences of $|\gamma'|$ and T_c. The γ' value was evaluated by fitting the form, $S/T = \gamma' \ln(T_0/T)$, to the experimental data. The T_c is the midpoint as described above.

Supplementary Note 6. γ' values of $S/T = \gamma' \ln(T_0/T)$ for other materials

Supplementary Table 1 lists the $|\gamma'|$ values reported in literatures[6-18], where γ' values were evaluated from the behaviour of $S/T = \gamma'\ln(T_0/T)$.

Supplementary Note 7. Estimation of the coefficient of *T*-square term in resistivity

The coefficient, A, in the form of $\rho=\rho_0+AT^2$ was evaluated by fitting the temperature dependence of the resistivity at low temperatures (Supplementary Figure 5a). The fitting temperature regions were represented in Supplementary Figure 5b.

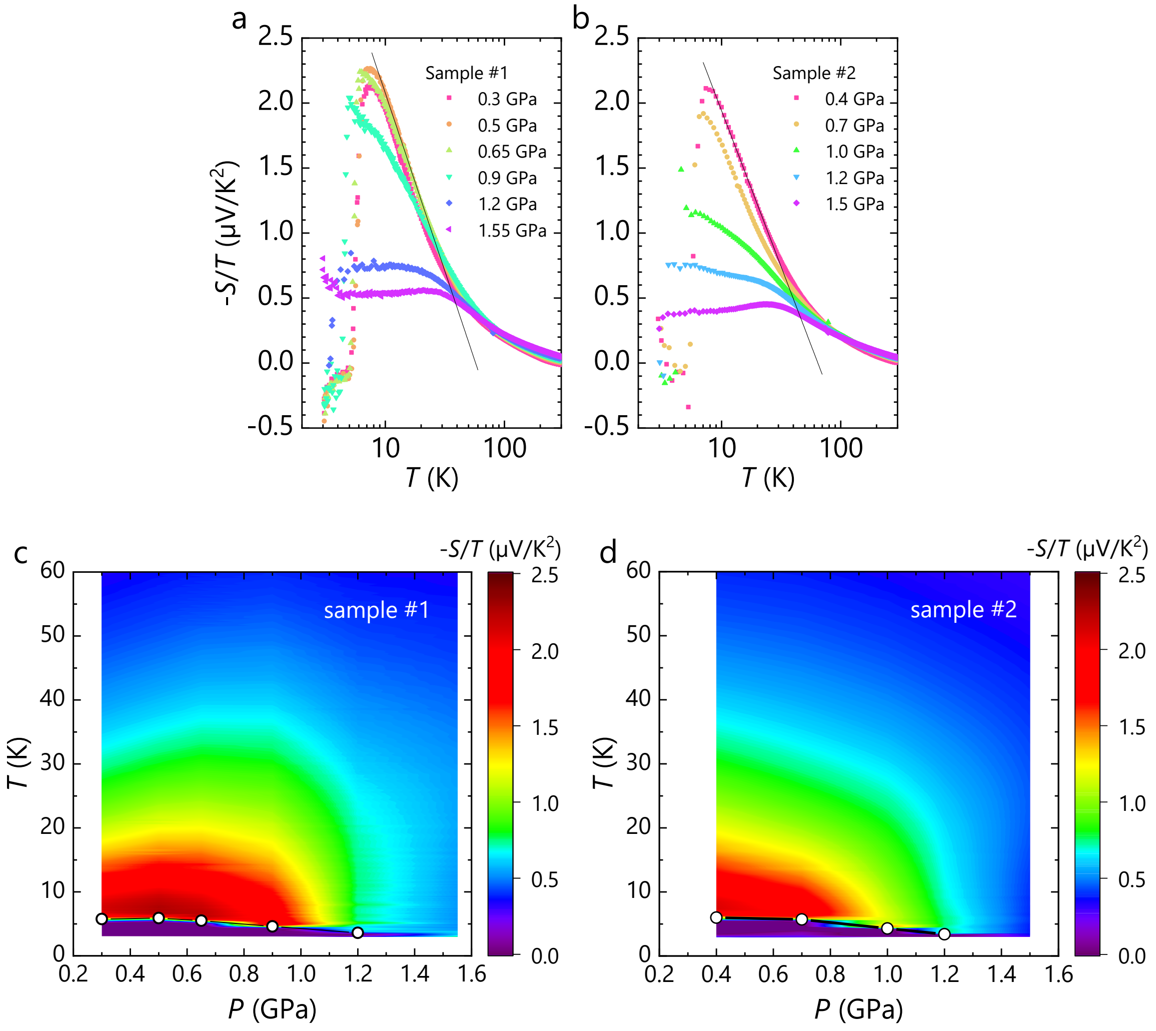

Supplementary Figure 1. Temperature dependence and the contour plot of -*S*/*T* in κ-HgBr for samples #1 and #2. The open circles in Supplementary Figures 1c and 1d indicate the superconducting transition temperature.

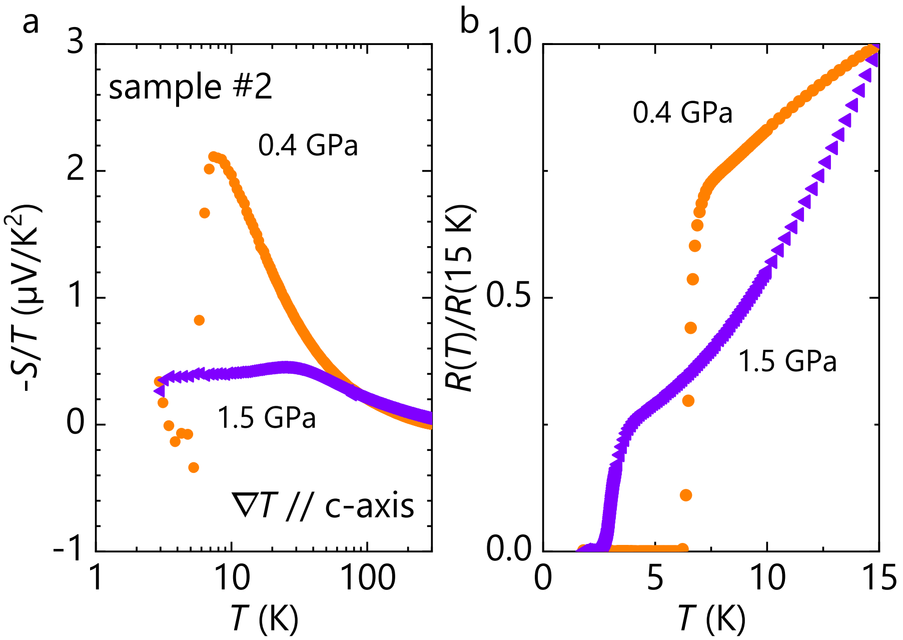

Supplementary Figure 2. Temperature dependences of the -*S*/*T* and resistivity simultaneously measured for sample #2 at 0.4 and 1.5 GPa. a, Temperature dependence of -*S*/*T* in the logarithmic temperature scale. At 0.4 GPa, -*S*/*T* exhibits ln*T* behaviour. At 1.5 GPa, however, the Fermi liquid behaviour of -*S*/*T*=constant was observed. **b**, Temperature dependence of the resistivity normalized to its value of 15 K. The linear- and quadratic-in-temperature resistivity above T_c under 0.4 and 1.5 GPa indicate non-Fermi liquid and Fermi liquid behaviours, respectively.

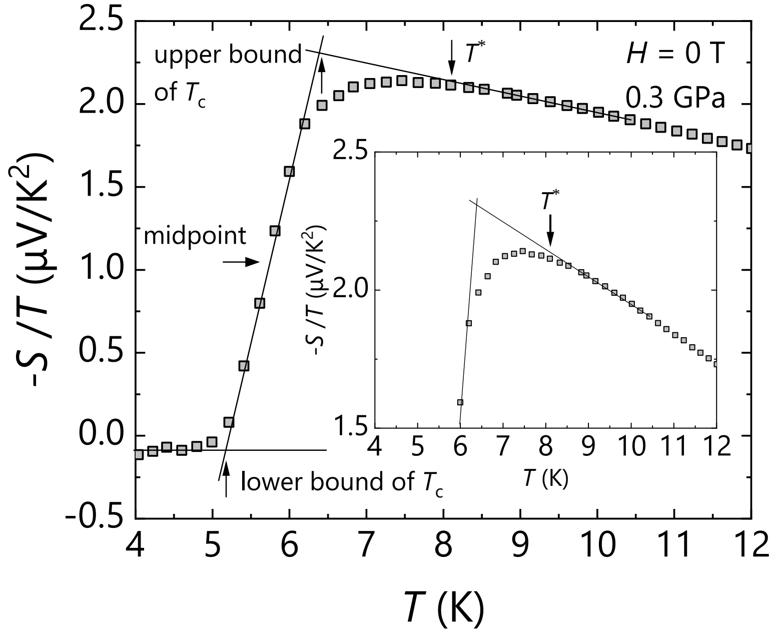

Supplementary Figure 3. Definition of T_c and T^*. The definitions of bulk T_c, its error, and the onset, T^*, are shown for the 0.3 GPa data of -S/T of sample #1, for example. The bulk T_c is defined by the midpoint. The upper and lower bounds of T_c indicated in the figure determine the error bar of T_c. T^* is defined as the temperature at which -S/T starts to deviate from the normal-state behaviour. The inset shows the enlarged view of the -S/T behaviour in the vicinity of T^*.

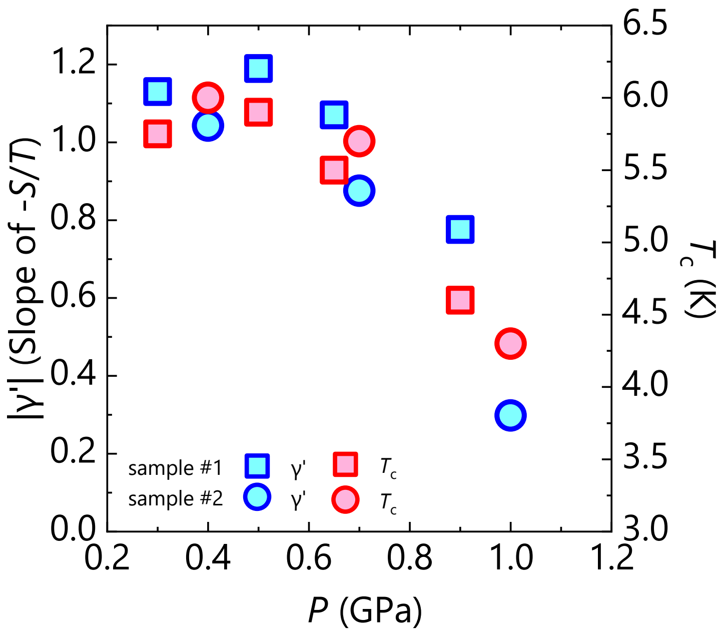

Supplementary Figure 4. Pressure dependence of |γ'| and T_c in κ-HgBr. The |γ'| and T_c values for samples #1 and #2 are plotted as a function of pressure. The squares and circles correspond to the data of samples #1 and #2, respectively. The blue and red symbols indicate |γ'| and T_c, respectively.

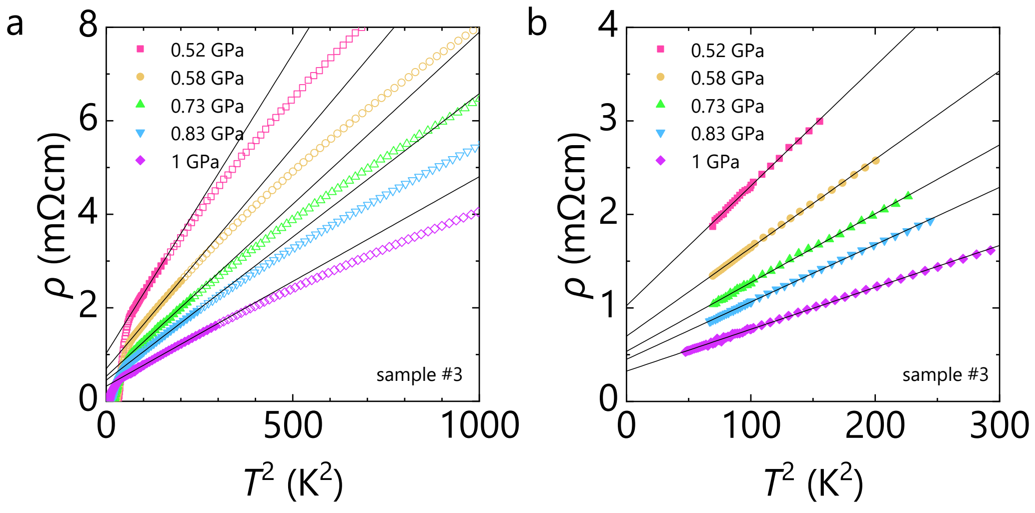

Supplementary Figure 5. Resistivity versus squared temperature in ranges of (a) 0-1000 K^2 and (b) 0-300 K^2. The resistivity ρ follows the Fermi liquid behaviour of $\rho = \rho_0 + AT^2$ at low temperatures and the values of the coefficient A plotted in the inset of Fig. 3a were determined from the data shown in Supplementary Figure 5b.

Supplementary Table 1. |γ'| values in the logarithmic part of -*S*/*T* in some materials. The |γ'| values in the behaviour of $S/T = \gamma' \ln(T_0/T)$ for other materials[6-18] are listed in μV/K^2.

Material	Slope value	Material	Slope value
$La_{1.6-x}Nd_{0.4}Sr_xCuO_4$ (Nd-LSCO (x = 0.24))(ref.[6])	0.11	UCoGe (*H*=11.1 T) (ref.[12])	2.3
$La_{1.8-x}Eu_{0.2}Sr_xCuO_4$ (Eu-LSCO (x = 0.24)) (ref.[7])	0.16	$YbRh_2Si_2$ (ref.[13])	4.5
$Bi_2Sr_{2-x}La_xCuO_{6+\delta}$ (Bi2201 (*p* = 0.39) (ref.[8])	0.05	$CeCu_{5.9}Au_{0.1}$ (ref.[14])	6.2
$Pr_{2-x}Ce_xCuO_4$ (PCCO (x = 0.16-0.19)) (ref.[9])	0.012-0.038	Ce_2PdIn_8 (ref.[15])	1.6
$La_{2-x}Ce_xCuO_4$ (LCCO (x = 0.15-0.17)) (ref.[9])	0.0095 – 0.049	YbAgGe (ref.[16])	4.7
$[BiBa_{0.66}K_{0.36}O_2]CoO_2$ (ref.[10])	0.62	YbPtBi (ref.[17])	0.25
$Ba(Fe_{1-x}Co_x)_2As_2$ (x=0.022-0.13) (ref.[11])	0.32 - 0.895	$EuFe_2(As_{1-y}P_y)_2$ (y=0.26, 0.36) (ref.[18])	0.077, 0.16

Supplementary References

1. Lyubovskaya, R. N. et al. Is the organic metal $(ET)_4Hg_3Br_8$ a Quasi-2d superconductor? *JETP Lett.* **45**, 530–533 (1987).
2. Li, R., Petricek, V., Yang, G., Coppens, P. & Naughton, M. Room- and Low-Temperature Crystallographic Study of the Ambient Pressure Organic Superconductor (Bisethylene dithiotetrathiofulvalene)$_4Hg_{2.89}Br_8$. *Chem. Mater.* **10**, 1521-1529 (1998).
3. Yamamoto, T. et al. Examination of the Charge-Sensitive Vibrational Modes in Bis(ethylenedithio)tetrathiafulvalene. *J. Phys. Chem. B* **109**, 15226-15235 (2005).
4. Gillespie, R. J. et al. The Preparation and Structure of Chain and Sheet Mercury Compounds. *Phil. Trans. R. Sot. Lond.* A **314**, 105 (1985).
5. Shimizu, Y., Maesato, M. & Saito, G. Uniaxial Strain Effects on Mott and Superconducting Transitions in κ-$(ET)_2Cu_2(CN)_3$. *J. Phys. Soc. Jpn.* **80**, 074702 (2011).
6. Daou, R. et al. Thermopower across the stripe critical point of $La_{1.6-x}Nd_{0.4}Sr_xCuO_4$: evidence for a quantum critical point in a hole-doped high-T_c superconductor. *Phys. Rev. B* **79**, 180505 (2009).
7. Laliberté, F. et al. Fermi-surface reconstruction by stripe order in cuprate superconductors. *Nature Commun.* **2**, 432 (2011).
8. Lizaire, M. et al. Transport signatures of the pseudogap critical point in the cuprate superconductor $Bi_2Sr_{2-x}La_xCuO_{6+\delta}$. *Phys. Rev. B* **104**, 014515 (2021).
9. Mandal, P. R., Sarkar, T. & Greene, R. L. Anomalous quantum criticality in the electron-doped cuprates. *Proc. Natl Acad. Sci. USA* **116**, 5991–5994 (2019).
10. Limelette, P., Saulquin, W., Muguerra, H. & Grebille, D., From quantum criticality to enhanced thermopower in strongly correlated layered cobalt oxide. *Phys. Rev. B* **81**, 115113 (2010).
11. Arsenijević, S. et al. Signatures of quantum criticality in the thermopower of $Ba(Fe_{1-x}Co_x)_2As_2$. *Phys. Rev. B* **87**, 224508 (2013).
12. Malone, L. et al. Thermoelectricity of the ferromagnetic superconductor UCoGe. *Phys. Rev. B* **85**, 024526 (2012).
13. Hartmann, S. et al. Thermopower evidence for an abrupt Fermi surface change at the quantum critical point of $YbRh_2Si_2$. *Phys. Rev. Lett.* **104**, 096401 (2010)
14. Kuwai, T. et al. Thermoelectric Power at Low Temperatures in $Ce(Ni_{1-x}Pd_x)_2Ge_2$ and $CeCu_{5.9}Au_{0.1}$ in the Vicinity of Antiferromagnetic Quantum Critical Point. *J. Phys. Soc. Jpn.* **80**, SA064 (2011).

15. Matusiak, M., Gnida, D. & Kaczorowski, D. Quantum criticality in Ce_2PdIn_8: A thermoelectric study. *Phys. Rev. B* **84**, 115110 (2011).
16. Mun, E. D., Bud'ko, S. L. & Canfield, P. C. Thermoelectric power investigations of YbAgGe across the quantum critical point. *Phys. Rev. B* **82**, 174403 (2010).
17. Mun, E. D. et al. Magnetic-field-tuned quantum criticality of the heavy fermion system YbPtBi. *Phys. Rev. B* **87**, 075120 (2013).
18. Maiwald, J., Jeevan, H. S. & Gegenwart, P. Signatures of quantum criticality in hole-doped and chemically pressurized $EuFe_2As_2$ single crystals. *Phys. Rev. B* **85**, 024511 (2012).
